# Editorial: Molecular and nanoscale engineering of nucleic acid theranostics and vaccines

**DOI:** 10.3389/fbioe.2022.1126876

**Published:** 2023-01-05

**Authors:** Xiaohong Tan, Zilong Zhao, Ruowen Wang, Guizhi Zhu

**Affiliations:** ^1^ Department of Chemistry and Center for Photochemical Sciences, Bowling Green State University, Bowling Green, OH, United States; ^2^ Molecular Science and Biomedicine Laboratory (MBL), State Key Laboratory of Chemo/Biosensing and Chemometrics, College of Chemistry and Chemical Engineering, Aptamer Engineering Center of Hunan Province, Hunan University, Changsha, China; ^3^ Institute of Molecular Medicine, Ren Ji Hospital, School of Medicine, Shanghai Jiao Tong University, Shanghai, China; ^4^ Department of Pharmaceutics and Center for Pharmaceutical Engineering and Sciences, School of Pharmacy, Virginia Commonwealth University, Richmond, United States

**Keywords:** synthetic nucleic acids, aptamer, mRNA vaccine, theranostics, diagnostics

Nucleic acids, such as antisense oligonucleotides (ASO), small interfering RNA (siRNA), miRNA, aptamers, plasmids, mRNA, have been widely explored as synthetic structural biomaterials and functional theranostics and vaccines. Among the above nucleic acids, aptamers are single-stranded oligonucleotides that specifically recognize and bind to various targets by forming unique structures, making them “chemical antibodies” in various applications. In another example, synthetic mRNA therapeutics and vaccines have been explored for the prevention of infectious diseases, such as COVID-19 and flu, and for the treatment of a variety of diseases ranging from cancer, cardiovascular diseases, to autoimmune diseases. This Research Topic, *Molecular and Nanoscale Engineering of Nucleic Acid Theranostics and Vaccines*, focuses on the most recent advances in this field. Specifically, we present a Research Topic of seven original research and review articles that provide a peek into the recent discoveries and innovations that may expedite the use of nucleic acids for disease diagnostics, prevention, and therapy.

Cardiovascular disease is a major cause of death worldwide, and novel diagnostic tools and treatments for this disease are urgently needed. Chemically synthesized aptamers have been engineered for the diagnosis and therapy of cardiac diseases. For example, a combination of von Willebrand Factor and aptamers can effectively inhibit the progression of blood clotting, presenting a positive diagnosis and therapeutic effect, and laying a novel strategy to improve the biocompatibility of paclitaxel drug balloon or implanted stent in the future. In this Research Topic, Chen et al. summarized aptamer-based applications in cardiovascular disease, including biomarker discovery and future management strategy. Although the related clinical applications remains to be further tested and validated so far, the significant research advancement have demonstrated that aptamers can be promising agents to realize the great potential in the diagnosis and therapy of cardiac diseases.

One of the most promising areas that aptamers’ application has been explored is *in vitro* and *in vivo* disease diagnosis. Among various approaches towards this end, aptamers have been studied for molecular imaging through imaging modalities such as positron emission tomography (PET) and single-photon emission tomography (SPECT). Liu, et al. presented a comprehensive summary of the radiolabeling methodology of aptamers and the resulting aptamer-based molecular imaging of biomarkers in a wide range of diseases. In addition to aptamers, these radiolabeling approaches can easily be adapted to label other oligonucleotide theranostics, such as siRNA and ASO.

Aptamers are also an appealing class of molecular ligands for targeted drug delivery, which has been widely explored in the therapy of diseases such as cancer and infectious diseases. Gao et al. summarize the clinical applications of aptamers and highlight the research in aptamer-drug conjugates and aptamer-based nanomaterial systems for targeted drug delivery. In this mini-review, the authors describe the perspectives, challenges, and opportunities in the field.

Tang et al. report the design and synthesis of a solid-phase module containing cycloastragenol (CAG), which is an efficient ingredient isolated from Chinese herbal. They demonstrate that CAG can be incorporated into oligonucleotide (ON) automatically by oligonucleotide synthesis technology. Furthermore, experiments confirm that ON-CAG conjugate retains the renoprotective effect as CAG. The research indicates that solid phase synthesis could be a unique technology for the mechanism study of Chinese Traditional Medicine.

Nucleic acid diagnostics have been extensively studied for the detection of disease-associated bioanalytes, such as viral genomic DNA or RNA. This was most recently exemplified by the detection for the severe acute respiratory disease coronavirus 2 (SARS-COV-2) viruses that cause COVID-19. Though polymerase chain reaction (PCR)-based SARS-COV-2 tests remain one of the most dominant approaches, this approach is also associated with limitations such as the need of sophisticated facilities and equipment, as well as the relatively long lead time. By contrast, as Ma et al. summarized, PCR-free approaches hold great potential to address these Research Topic for SARS-COV-2 viral nucleic acid detection. Specifically, these methods involve unique mechanisms such as isothermal nucleic acid amplification, nucleic acid enzymes, electrochemistry, and clustered regularly interspaced short palindromic repeats (CRISPR).

The development of mRNA vaccines have made a leap due to the vast success of COVID-19 mRNA vaccines. Guillermo Aquino-Jarquin briefly discusses the development of the mRNA vaccines in the concise communication and analyzes the critical aspects concerning the structural elements of the mRNA sequences used in the first two mRNA vaccines for COVID-19 (mRNA-1273 and BNT162b2), which will help understand the patent dispute between Moderna and Pfizer/BioNTech. Although the dispute seems more complex than previously thought, which might need serval years to reach the final patent decisions, the mRNA vaccine really provides a powerful kind of tools for disease treatment.

In the last research article, Xiang et al. contributed to the mechanistic understanding of how a long non-coding RNAs (lncRNAs), metastasis-associated lung adenocarcinoma transcript 1 (MALAT1), impacts the progression of non-alcoholic fatty liver disease (NAFLD). As exemplified by this study, such illustration of endogenous lncRNAs may uncover novel therapeutic targets and pave the way to the development of novel nucleic acid therapeutics for a variety of diseases.

## Summary

The articles presented in this Research Topic provide a synopsis of many of the most recent advancements in nucleic acid theranostics and vaccines. The Editors are delighted to highlight this work. This Research Topic can help provide a foundation for future studies, that could lead to the development of transformative new theranostics and vaccines.

